# Glycine Inhibitory Dysfunction Turns Touch into Pain through PKCgamma Interneurons

**DOI:** 10.1371/journal.pone.0001116

**Published:** 2007-11-07

**Authors:** Loïs S. Miraucourt, Radhouane Dallel, Daniel L. Voisin

**Affiliations:** 1 INSERM, E216, Clermont-Ferrand, F-63000 France; 2 Université Auvergne-Clermont1, Clermont-Ferrand, F-63000 France; 3 CHU Clermont-Ferrand, Clermont-Ferrand, F-63000 France; Columbia University, United States of America

## Abstract

Dynamic mechanical allodynia is a widespread and intractable symptom of neuropathic pain for which there is a lack of effective therapy. During tactile allodynia, activation of the sensory fibers which normally detect touch elicits pain. Here we provide a new behavioral investigation into the dynamic component of tactile allodynia that developed in rats after segmental removal of glycine inhibition. Using *in vivo* electrophysiological recordings, we show that in this condition innocuous mechanical stimuli could activate superficial dorsal horn nociceptive specific neurons. These neurons do not normally respond to touch. We anatomically show that the activation was mediated through a local circuit involving neurons expressing the gamma isoform of protein kinase C (PKCγ). Selective inhibition of PKCγ as well as selective blockade of glutamate NMDA receptors in the superficial dorsal horn prevented both activation of the circuit and allodynia. Thus, our data demonstrates that a normally inactive circuit in the dorsal horn can be recruited to convert touch into pain. It also provides evidence that glycine inhibitory dysfunction gates tactile input to nociceptive specific neurons through PKCγ-dependent activation of a local, excitatory, NMDA receptor-dependent, circuit. As a consequence of these findings, we suggest that pharmacological inhibition of PKCγ might provide a new tool for alleviating allodynia in the clinical setting.

## Introduction

Neuropathic pain is due to lesion or dysfunction of the peripheral or central nervous system, which generates and maintains abnormal, increased neuronal sensitivity [1]. It presents a major therapeutic challenge to healthcare professionals since it is one of the most difficult syndromes to treat successfully [2]. However, a new concept has been proposed, in which pain symptoms are analyzed on the basis of underlying mechanisms [3]. Increased knowledge of pain-generating mechanisms and their translation into symptoms should allow a dissection of the mechanisms that are at play in each patient [4], [5]. This, combined with a selection of drugs that act on those mechanisms should make it possible to design optimal treatments for individual patients [6]. Here, we investigated the mechanisms of dynamic mechanical allodynia, one hallmark and disabling symptom of neuropathic pain.

Dynamic mechanical allodynia is pain produced by normally non-painful light-pressure moving stimuli on skin [1]. It is established that dynamic mechanical allodynia is mediated by peripheral low-threshold, large myelinated Aβ-fibers [7]–[9]. These sensory fibers normally do not produce pain and are responsible for the detection of innocuous mechanical stimuli only. After nerve damage, however, activation of these afferents elicits pain. Past research has shown that the mechanical allodynia that occurs after peripheral nerve injury depends on the hyperexcitability of neurons in the dorsal horn of the spinal cord too [10]. Although such increased neuronal sensitivity involves excitatory synaptic mechanisms, recent findings emphasize that disinhibition through reduced inhibitory transmitter synthesis and/or release [11], [12], loss of inhibitory interneurons [13], shift in anion gradient [14], [15] or altered descending inhibitory modulation from the brain [16] can also dramatically alter the excitability of pain transmission neurons after nerve injury. Inhibitory glycine receptors and glycinergic neurons are abundant in the dorsal horn [17], [18] and thus significant disinhibition may occur following alterations in glycine-mediated inhibition. Accordingly, animal studies showed that blockade of strychnine-sensitive glycine receptors within the spinal cord results in profound tactile allodynia [19]–[21] and pain in response to light touch also develops in human during strychnine intoxication [22]. Furthermore, glycine receptors are reduced in number within segmental gray matter in a model of neuropathic pain [23]. Thus, in the present work we investigated the mechanisms of dynamic mechanical allodynia following segmental removal of glycine inhibition.

In contrast to dynamic mechanical allodynia, physiological pain initiates from primary sensory neurons called nociceptors [24]. These include thin unmyelinated C-fibers and myelinated Aδ-fibers, whose central terminals make synaptic contact with second order neurons that are at the origin of pain-related pathways [25]. Nociceptors contact nociceptive-specific (NS) neurons that respond to nociceptive stimuli only and are located in superficial laminae (I-II) of the dorsal horn. They also activate through mono- or polysynaptic pathways wide dynamic range (WDR) nociceptive neurons that are located mainly in deep lamina (V) of the dorsal horn. In contrast to NS neurons, WDR neurons also respond to innocuous peripheral stimuli since they receive direct input from peripheral non-nociceptive large myelinated Aβ-fibers [10]. However, there is evidence for low threshold C fiber input to superficial laminae [26]–[29] and polysynaptic Aβ fiber responses in lamina I putative NS neurons have been reported after disinhibition [30]. To decipher the mechanisms of dynamic mechanical allodynia, a crucial question is to understand how sensory processing is altered in the dorsal horn after disinhibition, to such an extent that activation of Aβ fibres elicits pain.

In this study, we wished to test the hypothesis that disinhibition underlies dynamic mechanical allodynia by gating Aβ input to WDR nociceptive neurons. We also tested the alternative hypothesis that disinhibition opens a physiologically silent path that can take tactile information to superficial NS neurons. We used the trigeminal system as a model, since dynamic mechanical allodynia is extremely frequent in trigeminal neuropathic syndromes [31]. We finally sought to determine the cellular elements involved in the process.

## Results

### Segmental disinhibition induces tactile allodynia

**Figure 1:**
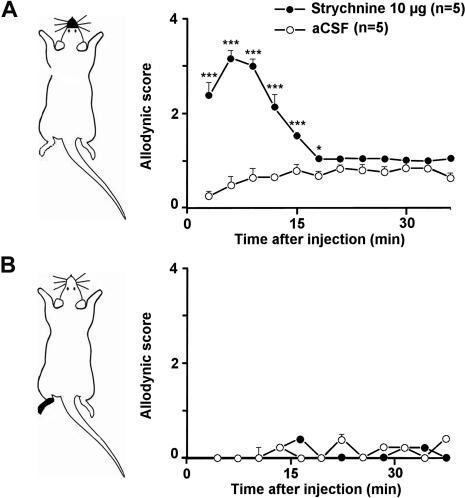
Trigeminal disinhibition induces facial tactile allodynia. (A) Time course of changes in behavioral responses evoked by dynamic tactile mechanical stimulation (air puff) of the face of intracisternaly strychnine injected rats and aCSF injected rats. (B) Time course of changes in behavioral responses evoked by dynamic tactile mechanical stimulation (air puff) of the hindpaw of intracisternaly strychnine injected rats and aCSF injected rats. Graphs represent the mean response (±s.e.m.) of rats to 5 gentle air puffs applied at 10 s intervals. *, P<0.05; ***, P<0.001, strychnine *vs.* aCSF.

Pharmacological removal of trigeminal glycine inhibition was obtained by blockade of segmental glycine receptors by the glycine receptor antagonist strychnine [32]. Strychnine (10 µg dissolved in 5 µl artificial cerebrospinal fluid, aCSF) was injected into the cisterna magna under brief (<3 minutes) halothane anesthesia. Rats quickly recovered from anesthesia (<2 minutes) and then were left freely moving in an open field under red light for 36 minutes. Their responses to light, dynamic mechanical stimulations (gentle air puffing) either onto the face or onto the hindpaw were scored every 3 minutes, based on the number of observed response elements that were assumed to reflect the nociceptive quality of the evoked sensation [33]. Intracisternal injection of strychnine (10 µg in 5 µl aCSF) produced a dramatic face allodynia with the highest mean score reaching 3.2±0.2 (n = 5) on a scale ranging from 0 to 4 (Figure 1A). Allodynia started immediately after the injection and lasted for 18 minutes. During this period, scores were significantly different (p<0.05-0.001) from those of control animals that received aCSF only (n = 5). Scores in control rats were similar to those in animals that were not anesthetized and not injected (data not shown). During the whole testing procedure the response scores to stimulation of the hindpaw were close to zero and not different in the strychnine (n = 5) and control groups (n = 5; Figure 1B). Since the mechanical allodynia provoked by intracisternal strychnine was localized to the face, it is probable that glycine receptors were blocked at the trigeminal level only.

### Disinhibition of WDR neurons does not underlie allodynia

**Figure 2:**
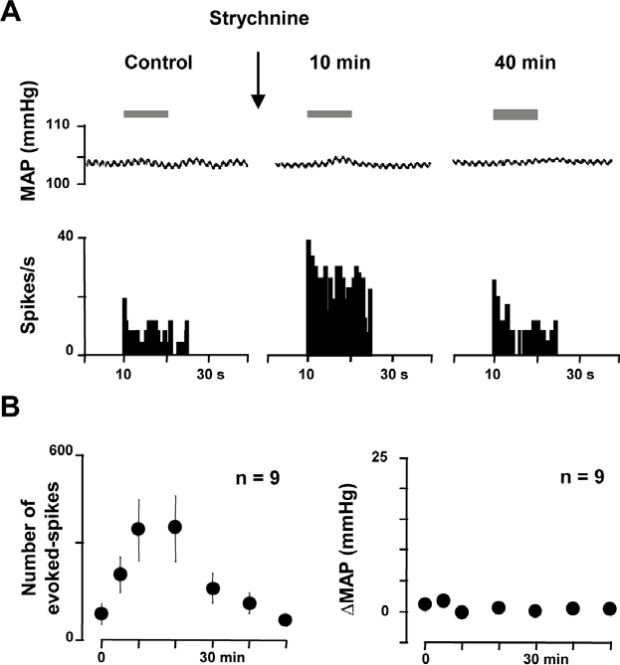
Direct disinhibition of WDR neurons does not underlie allodynia. (A) Example of the effects of strychnine applied within the Sp5O on responses to light brushing of WDR neuron receptive field (bar). The simultaneous mean arterial blood pressure recording (MAP) is shown on the top of the trace. (B) Graphs illustrating the time-course of changes in firing of Sp5O WDR neurons (left) and the time-course of changes in blood pressure (ΔMAP, right) in response to innocuous Aβ- mediated stimuli (light brushing) after strychnine application within the Sp5O. Results are expressed as mean±SEM, n refers to the number of animals tested (only one neuron per animal was tested). Although WDR neurons displayed higher firing in response to innocuous stimuli after strychnine, blood pressure remained stable.

Since among second order nociceptive neurons, only WDR neurons receive innocuous input from Aβ primary sensory fibers, we hypothesized that disinhibition may induce allodynia by increasing responses in these neurons. The responses of trigeminal WDR neurons to light dynamic mechanical stimulations under segmental glycine receptor blockade were thus investigated in halothane-anesthetized, curarized and ventilated rats. The significance of the changes in the responses of central trigeminal neurons was examined by correlating them with the occurrence of the simultaneously recorded cardiovascular responses [34]. In the rat trigeminal sensory system, an important pool of WDR nociceptive neurons can be easily recorded from the subnucleus oralis (Sp5O) [35], [36], where NS neurons are rare. We thus recorded and tested a total of 16 WDR neurons from the Sp5O. They were not spontaneously active. As previously shown [35], when we applied percutaneous electrical stimuli at threshold for C-fiber activation to the center of the excitatory receptive field of all WDR neurons, responses attributable to peripheral activation of A- and C-fibers could be observed. The mean minimal latency of the first burst of action potentials was 2.17±0.08 ms (2–2.5 range). Based on the approximate distance between the stimulating electrode and the trigeminal neurons, the conduction velocity was ∼43 m/sec, which corresponds to Aβ-fiber conduction velocity. Accordingly, in response to receptive field stimulation known to engage Aβ-fibers only, such as light sweeping with a soft paintbrush, all neurons fired brief bursts of action potentials (Figure 2A). They also increased firing rates as the intensity of the stimuli increased into the nociceptive range.

Micro-infusion of strychnine (0.5 µg in 0.25 µl aCSF) in the Sp5O significantly increased firing responses of WDR neurons to brushing (n = 9), without altering their basal firing and this effect lasted for 29±3 minutes (Figure 2). However, the blood pressure of the animal remained unchanged during Sp5O strychnine application and peripheral stimulations as well, both before and after strychnine (Figure 2). Since changes in blood pressure provide a reliable index of nociception in anaesthetized animals [34], innocuous stimuli were not regarded as nociceptive under these circumstances, although WDR neurons displayed higher firing responses.

**Figure 3:**
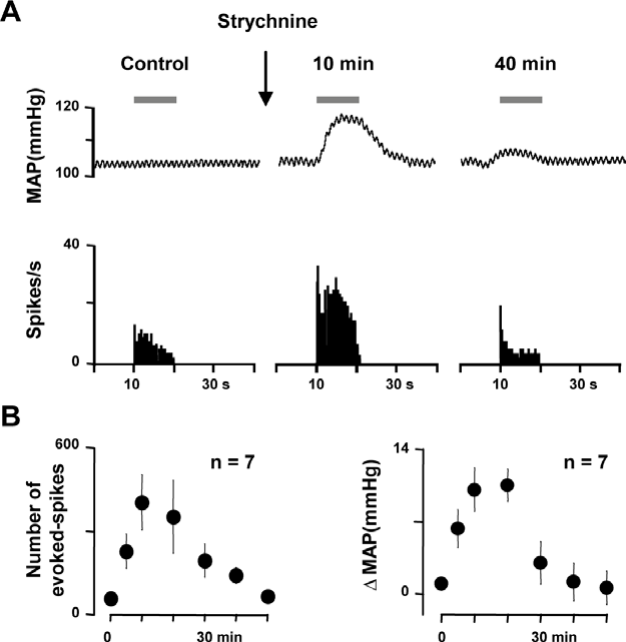
Strychnine administered within the superficial MDH disinhibits tactile activities in the Sp5O. (A) Example of the effects of strychnine applied within the superficial MDH on responses of a Sp5O WDR neuron to light brushing of its receptive field (bar). The simultaneous mean arterial blood pressure recording (MAP) is shown on the top of the trace. (B) Graphs illustrating the time-course of changes in firing of Sp5O WDR neurons (left) and the time-course of changes in blood pressure (ΔMAP, right) in response to innocuous Aβ- mediated stimuli (light brushing) after strychnine application within the superficial MDH. Results are expressed as mean±SEM, n refers to the number of animals tested (only one neuron per animal was tested). WDR neurons displayed higher firing in response to innocuous stimuli after strychnine application within the superficial MDH and this was paralleled with reversible increases in blood pressure.

We have demonstrated previously that Sp5O WDR neurons receive C-fiber inputs indirectly, through relay interneurons located within the superficial medullary dorsal horn (MDH) laminae [35], [37]. We thus asked whether disinhibition of this connection would affect the capacity of Sp5O WDR neurons to respond to light tactile stimuli. Micro-infusion of strychnine (0.5 µgin 0.25 µlaCSF) within the superficial laminae of the MDH (n = 7) significantly increased the firing responses of Sp5O WDR neurons to brushing (Figure 3). The effect of strychnine lasted for 31±3 minutes and was paralleled with significant and reversible increases in blood pressure in response to innocuous stimuli (Figure 3B). Thus, when glycine inhibition was blocked within the superficial laminae, dynamic tactile stimuli were regarded as nociceptive.

### Disinhibition allows touch activation of nociceptive specific neurons

**Figure 4:**
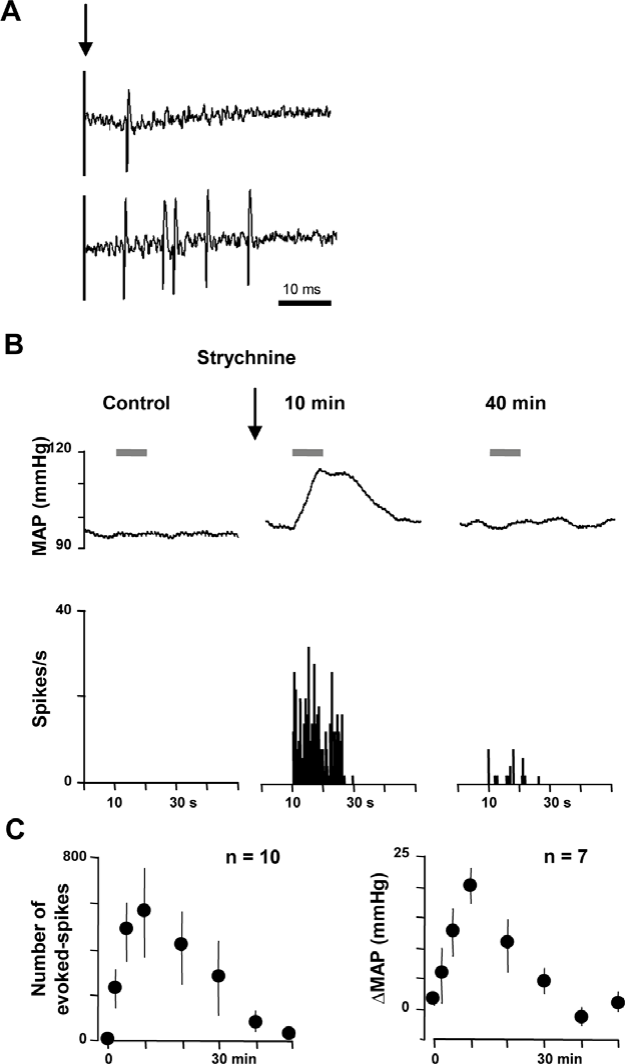
Disinhibition allows touch activation of NS neurons. (A) Original trace of the neural responses to electrical stimulation (arrow) of the receptive field of a superficial lamina NS neuron. The stimulus elicited a response (9 ms) within the Aδ-fiber range. (B) Example of the effects of intracisternal strychnine on responses of a superficial lamina NS neuron to light brushing of its receptive field (bar). The simultaneous mean arterial blood pressure recording (MAP) is shown on the top of the trace. (C) Graphs illustrating the time-course of changes in firing of superficial lamina NS neurons (left) and the time-course of changes in blood pressure (ΔMAP, right) in response to innocuous Aβ- mediated stimuli (light brushing) after intracisternal strychnine injection. Results are expressed as mean±SEM, n refers to the number of animals tested (only one neuron per animal was tested). Previously unresponsive NS neurons were activated by innocuous stimuli after strychnine and this was paralleled with reversible increases in blood pressure.

Since only indirect, but not direct disinhibition of WDR neurons induced nociceptive responses to dynamic tactile stimuli, we investigated whether disinhibition by strychnine in the superficial laminae of the MDH could allow touch activation of NS neurons. We recorded and tested a total of 10 NS neurons from the superficial laminae of the MDH of halothane-anesthetized, curarized and ventilated rats. All neurons were classified as NS because they were excited by nociceptive mechanical stimuli applied to their cutaneous receptive field, but not by innocuous tactile stimuli. In all cases, nociceptive stimuli only induced parallel and reversible increases in blood pressure. When we applied percutaneous electrical stimuli at threshold for C-fiber activation to the center of the excitatory field of the neurons, responses attributable to peripheral activation of Aδ- and/or C-fibers could be observed (Figure 4A). The mean minimal latency of the first burst was 7.7±0.3 ms (7–10 range, n = 10). Based on an approximate distance between the stimulating electrode and the trigeminal neurons, the conductance velocity was ∼7 m/sec, which corresponds to Aδ-fibers.

Accordingly, none of the trigeminal NS neurons we recorded from the superficial laminae of the MDH responded to receptive field stimulations known to engage Aβ-fibers only, such as light sweeping with a soft paintbrush (Figure 4B). However, after intracisternal strychnine injections (10 µg in 5 µl aCSF), innocuous mechanical stimuli of their receptive field by light brushing at this time activated all previously unresponsive NS neurons (Figure 4B). In these experiments, we did not perform direct micro-ejection of strychnine within the superficial laminae of the MDH, due to the technical difficulty of simultaneously recording from superficial lamina neurons. However, since the distance between the superficial laminae and the cisternal cavity is around 150 µm at the level recordings were obtained, it is likely that strychnine injected into the *cisterna magna* quickly diffused into the superficial laminae of the MDH to affect NS neuron firing. The effect of strychnine lasted for 37±4 minutes and was paralleled with significant and reversible increases in blood pressure in response to innocuous stimuli (Figure 4C). Under these circumstances thus, dynamic tactile stimuli were regarded as nociceptive and were rendered capable of activating normally unresponsive NS neurons.

Finally, we investigated whether disinhibition could unmask direct Aβ input to NS neurons. We found that NS neuron responses to electrical stimulation never occurred at latencies shorter than 7 ms after strychnine (n = 4, not shown). Thus, the responses evoked following disinhibition did not involve monosynaptic Aβ input to NS neurons.

### Disinhibition wakes up a normally silent circuit

**Figure 5:**
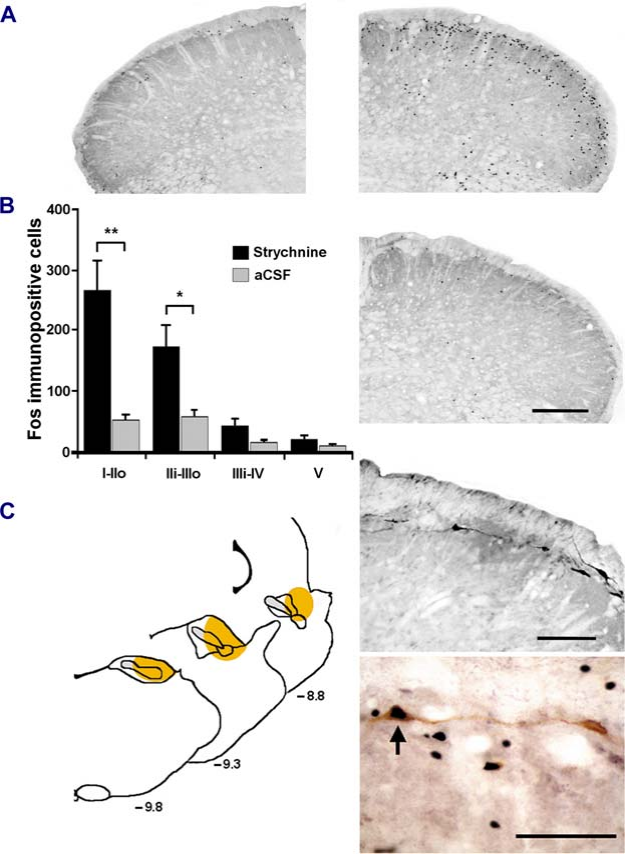
Fos expression reveals what dorsal horn neurons are activated during allodynia. (A) Images of Fos positive cell nuclei in the MDH; Fos was expressed following light brushing of the ipsilateral lip after intracisternal strychnine (top right). Contralateral side of the MDH in the same animal is shown on top left. Only sparse Fos positive cell nuclei were found in the MDH following light brushing of the lip after intracisternal aCSF (bottom right). Bar: 200 µm. (B) Bar histograms summarizing mean Fos expression in the different laminae of the ipsilateral MDH following light brushing of the lip after intracisternal strychnine or aCSF (n = 5/group). I-IIo: laminae I and outer II; IIi-IIIo: laminae inner II and outer III; IIIi-IV: laminae inner III and IV; V: lamina V. *, P<0.05; **, P<0.01, strychnine *vs.* aCSF. (C) Camera lucida diagrams representing the coronal level at which the retrograde tracer fluorogold deposition was maximal in the parabrachial area of 3 rats (left). Image of lamina I retrogradely labeled neurons in the MDH (top right; bar: 100 µm) and image of a retrogradely labeled neuron expressing Fos (bottom right, arrow; bar: 40 µm).

To map the neuronal circuit activated by innocuous mechanical stimulation under glycine receptor blockade, we used Fos expression as a surrogate measure of activity-evoked changes [38]. After intracisternal injection of strychnine (40 µg in 5 µl aCSF) in urethane anesthetized rats, light sweeping of the upper lip with a soft paintbrush (10 minutes, 0.5 Hz) resulted in strong, ipsilateral Fos expression in the superficial laminae of the MDH (Figure 5A), but not in the deep laminae, not in the Sp5O (not shown) and not in the contralateral side. Fos expression was predominant in lamina I, in the most superficial outer part of lamina II (laminae I-IIo), in the inner part of lamina II (IIi) and in the outer part of lamina III (IIIo; Figure 5B). In contrast, only minor expression was found in the MDH of control animals that received aCSF without strychnine before stimulation (Figure 5A). Thus, brush induced Fos-expression under pharmacological removal of glycine inhibition revealed which dorsal horn neurons may be responsible for allodynia.

We then ascertained whether part of these neurons were second order neurons projecting to upper brain structures. We thus combined retrograde tracing from the parabrachial area, a paramount structure integrating nociceptive information in the rat [39], with Fos expression in allodynic conditions. As previously reported [40], after injection of the retrograde tracer Fluorogold in the lateral parabrachial area, a number of retrogradely labeled cells were found in the superficial laminae of the MDH (Figure 5C). In addition, a significant proportion of double-labeled neurons were counted in lamina I, representing 15±4% (n = 3 rats) of retrogradely labeled neurons in lamina I (Figure 5C). Together with electrophysiological results, this data demonstrates that a simple switch in segmental glycine synaptic inhibition may turn touch into pain by unmasking the activation of superficial dorsal horn NS neurons by tactile stimuli.

However, low-threshold Aβ-fibers do not project to the very superficial laminae in the rat and thus cannot activate NS neurons directly. They reach the cellular region that overlaps the border between the inner part of lamina II and lamina III [25], [41]. The simultaneous expression of Fos in this region and in lamina I suggested the existence of a circuit that may convey non-nociceptive information from here to superficial NS neurons when it is disinhibited. Accordingly, the cellular region that overlaps the border between the inner part of lamina II and lamina III contains a substantial number of local dorsal horn interneurons, which themselves may activate NS neurons [25], most likely through polysynaptic connections [30]. One critical question was then to reveal the phenotypic identity of the circuit elements.

### The circuit involves PKCγ interneurons

**Figure 6:**
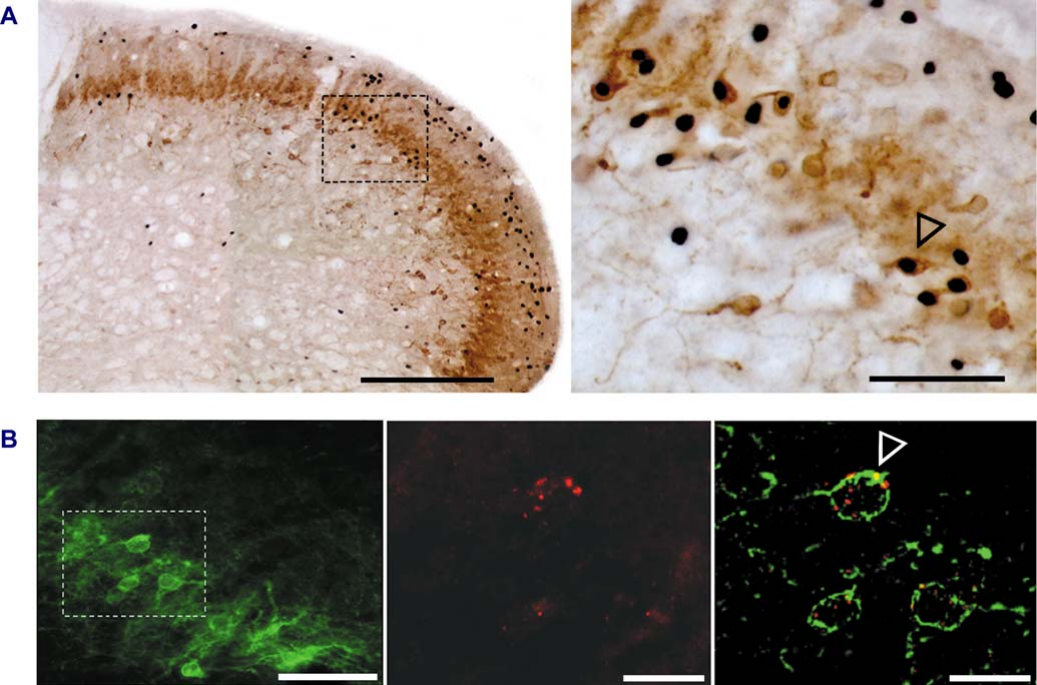
PKCγ expressing dorsal horn neurons are activated during allodynia. (A) Image of PKCγ immunopositive neurons and Fos positive cell nuclei in the MDH; bar: 200 µm. Fos was expressed following light brushing of the ipsilateral lip after intracisternal strychnine. The image on the right shows high power view of dual labeled neurons (arrowhead shows one typical example) located in the inner part of lamina II and in the outer part of lamina III; bar: 50 µm. (B) Fluorescence images of PKCγ immunopositive neurons (left, bar: 25 µm), glycine receptor α-β subunit immunoreactivity (middle, bar: 10 µm) and dual labeled neurons (right, arrowhead shows one example, bar: 10 µm).

A subset of dorsal horn interneurons critical for nociceptive information processing is selectively located in the inner part of lamina II and in the outer part of lamina III. They are characterized by expression of the gamma isoform of protein kinase C (PKCγ) [42], an important contributor to the increased pain sensitivity that occurs after injury [42], [43]. We thus asked whether Fos expressing neurons in allodynic conditions could belong to this population of interneurons. Using double immunocytochemical labeling, we found that 29±6% (n = 3) Fos expressing neurons located in the inner part of lamina II and in the outer part of lamina III of the MDH were also immunopositive for PKCγ (Figure 6A). Further double immunocytochemical labeling using an antibody directed against the glycine receptor α and β subunits showed that 30±1% (n = 3) PKCγ immunopositive interneurons in the inner part of lamina II and in the outer part of lamina III also express glycine receptors (Figure 6B). Thus, PKCγ immunopositive neurons potentially receive glycine-receptor mediated inhibition. These results suggest that the circuit that turns touch into pain when glycine inhibition is blocked involves dorsal horn interneurons expressing PKCγ.

### Selective PKCγ inhibition prevents allodynia

**Figure 7:**
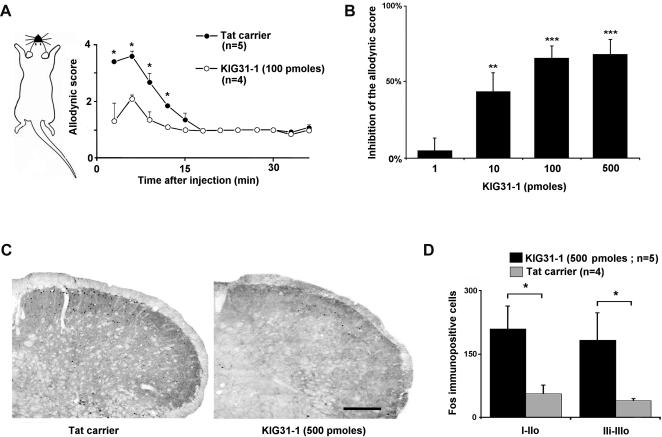
Selective PKCγ inhibition prevents allodynia and dorsal horn neuron activation. (A) Time course of changes in responses evoked by air puff applied on the face of intracisternaly strychnine injected rats after intracisternal administration of the selective PKCγ inhibitor KIG31-1 or of vehicle (Tat-carrier). KIG31-1 or vehicle was injected 30 minutes before strychnine; *, P<0.05. (B) Bar histogram illustrating the dose-dependent antiallodynic effect of intracisternal KIG31-1 (n = 4-5/group). Data is given as a percentage of control values obtained with injection of vehicle (Tat-carrier). **, P<0.01; ***, P<0.001, KIG31-1 *vs.* Tat carrier. (C) Examples of Fos immunolabeling in the MDH following light brushing of the ipsilateral lip after intracisternal strychnine in a control animal (Tat carrier, left) and in a 500 pmole KIG31-1 treated rat (KIG31-1, right). Bar is 200 µm. (D) Intracisternal administration of the selective PKCγ inhibitor KIG31-1 (500 pmoles) 30 minutes before strychnine resulted in a significant reduction in the brush-evoked Fos expression in the superficial laminae of the MDH. I-IIo: laminae I and outer II; IIi-IIIo: laminae inner II and outer III. *, P<0.05, KIG31-1 *vs.* Tat carrier.

We next asked whether PKCγ activation is a critical step in behavioral response and trigeminal nociceptive neuron excitation in the present model of trigeminal allodynia. To test this hypothesis, we used a recently developed, selective inhibitor of the gamma isoform of PKC (KIG31-1) [44]–[47]. First, intracisternal administration of KIG31-1 (dissolved in 5 µl vehicle, Tat carrier peptide) 30 minutes before strychnine injection (10 µg in 5 µl aCSF) resulted in a dose-dependent reduction in the scores of nociceptive responses of up to about 68% (Figure 7A, B). The ED50 value for reducing the score was 42.6 pmoles (95% confidence interval, 17.1-106.1 pmoles). Preemptive intracisternal injection of Tat carrier peptide alone did not change the score of nociceptive responses after strychnine (Figure 7A, B). Intracisternal injection of KIG31-1 (500 pmoles in 5 µl vehicle) did not affect motor performance of the animals, which was tested with rotarod (Table 1).

**Table 1:** Psychomotor effects of KIG31-1 assessed by the rotarod test.

Before treatment	Time after intracisternal KIG31-1 administration (500 pmoles)		
	30 min	50 min	60 min
224±47	233±39	270±23	229±43

Values are presented as mean±SEM (s, n = 4).

We were not able to test the effect of KIG31-1 on strychnine-induced touch-activation of NS neuron electrical activity, due to the extreme difficulty of recording from superficial lamina MDH neurons while carrying out several intracisternal injections. However, we examined whether KIG31-1 had non-selective effects on neuronal excitability. We found that KIG31-1 micro-infused within the Sp5O (100 pmoles in 5 µl vehicle) did not alter the firing response of Sp5O WDR neurons to non-nociceptive mechanical and electrical stimuli (n = 6, not shown).

Finally, we studied the effects of selective inhibition of PKCγ on brush-induced Fos expression after intracisternal injection of strychnine. We found that intracisternal administration of KIG31-1 (500 pmoles in 5 µl vehicle, n = 5) 30 minutes before the strychnine injection (40 µg in 5 µl aCSF) resulted in a dramatic reduction in brush-induced Fos expression in the MDH of urethane-anesthetized rats (Figure 7C, D). Compared to control animals that received preemptive intracisternal injection of vehicle alone (Tat carrier peptide, n = 4), Fos expression was reduced by 73±17% and 79±19% in lamina I-IIo and in the cellular region that overlap the border between lamina II and III respectively (P<0.05). These results show that selective inhibition of PKCγ in the dorsal horn can prevent the development of strychnine-induced tactile allodynia and activation of the underlying dorsal horn circuit as well.

### NMDA receptor blockade prevents allodynia

**Figure 8:**
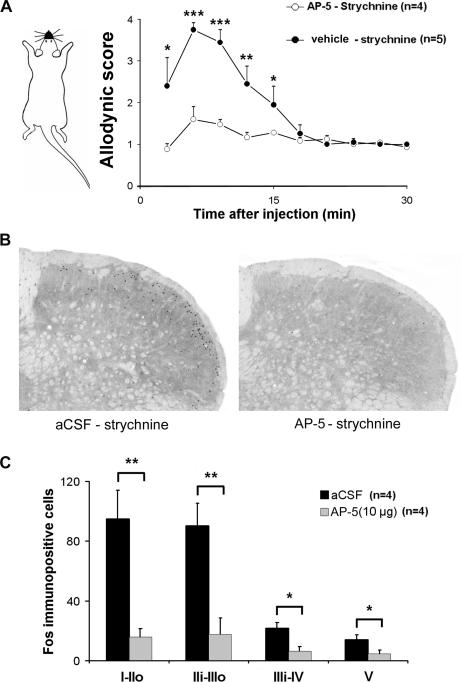
NMDA receptor blockade prevents allodynia and dorsal horn neuron activation. (A) Time course of changes in responses evoked by air puff applied on the face of intracisternaly strychnine injected rats after intracisternal administration of the selective NMDA receptor antagonist d-AP5 or of aCSF. d-AP5 or aCSF was injected 1 hour before strychnine; *, P<0.05. (B) Examples of Fos immunolabeling in the MDH following light brushing of the ipsilateral lip after intracisternal strychnine in a control animal (aCSF, left) and in a d-AP5 treated rat (AP-5, right). Bar is 200 µm. (C) Intracisternal administration of d-AP5 (10 µg in 5 µl aCSF) 1 hour before strychnine resulted in a significant reduction in the brush-evoked Fos expression in the superficial laminae of the MDH. I-IIo: laminae I and outer II; IIi-IIIo: laminae inner II and outer III. *, P<0.05, d-AP5 *vs.* aCSF.

PKCγ has been shown to contribute to a subset of the *N*-methyl-d-aspartate (NMDA)-dependent dorsal horn circuits that underlie injury-induced persistent pain [43]. It has also been demonstrated consistently that NMDA receptor activation is required for central sensitization in the dorsal horn [48]. We thus hypothesized that brush-activation of PKCγ under strychnine opened the gate to signaling in superficial dorsal horn neurons through an NMDA receptor-dependent excitatory circuit. To test the hypothesis, we used d-2-amino-5-phosphonovalerate (d-AP5), a selective antagonist of NMDA glutamate receptor [49]. Firstly, intracisternal administration of d-AP5 (10 µg in 5 µl aCSF) 1 hour before the strychnine injection (10 µg in 5 µl aCSF) dramatically reduced the behavioral scores of nociceptive responses to air puffs (Figure 8A). Secondly, intracisternal administration of d-AP5 (10 µg dissolved in 5 µl aCSF, n = 4) 1 hour before the strychnine injection (40 µg in 5 µl aCSF) and brushing of the lip resulted in a dramatic reduction in brush-induced Fos expression in the MDH of urethane-anesthetized rats (Figure 8C). By comparison with control animals that received preemptive intracisternal injection of aCSF alone (n = 4), Fos expression was reduced by 83±6% and 80±12% in lamina I-IIo and in the cellular region that overlap the border between lamina II and III respectively (P<0.05). These results show that blockade of NMDA receptors in the medullary dorsal horn can prevent development of strychnine-induced trigeminal tactile allodynia and activation of the underlying dorsal horn circuit as well.

## Discussion

In this study, we first provided a new behavioral investigation of the dynamic component of trigeminal mechanical allodynia that developed in rats after segmental removal of glycine inhibition. We found that in this condition, unmasking innocuous input to superficial dorsal horn NS neurons, as opposed to facilitation of WDR neurons, was mainly responsible for the strychnine-induced allodynia. We further sought to determine the cellular elements involved in the process. The activation was mediated through a local circuit involving neurons expressing PKCγ. Selective inhibition of PKCγ as well as antagonism of NMDA receptors in the dorsal horn prevented both activation of the circuit and allodynia. This data demonstrates that a normally inactive circuit in the dorsal horn can simply be recruited to convert touch into pain. It also shows that glycine inhibitory dysfunction gates Aβ input to NS neurons through PKCγ-dependent activation of a local, excitatory, NMDA receptor-dependent, circuit. As a consequence, pharmacological inhibition of PKCγ might provide a new tool for alleviating dynamic mechanical allodynia in the clinical setting.

### Disinhibition in the trigeminal brainstem system

In this study, we used the trigeminal system as a model, since dynamic mechanical allodynia is extremely frequent in trigeminal neuropathic syndromes. For instance, innocuous stimuli such as touch, hair movement or chewing are effective triggers for eliciting attacks of trigeminal neuralgia [31]. Furthermore, alteration of segmental inhibitory mechanisms has been involved in the pathogenesis of trigeminal neuralgia [50]. We took advantage of the anatomical organization of the rat spinal trigeminal nucleus to study the effects of glycine disinhibition on NS and WDR neuron activity. In the spinal trigeminal nucleus, a pool of WDR neurons is located rostrally, in the Sp5O, about 3 mm apart from the superficial laminae of the MDH where the NS neurons are situated [36], [51]–[56]. This allows local pharmacological applications to be carried out either in the MDH or in the Sp5O without directly affecting the other structure [35], [53]. Although the MDH is generally considered as the essential brainstem relay of orofacial nociceptive information [54], earlier findings have established the presence of nociceptive neurons in the Sp5O, the majority of which are WDR neurons [52], [55], [56]. The properties of Sp5O WDR neurons clearly match those of WDR neurons in lamina V of the medullary and spinal dorsal horn [36], [56]–[60]. Finally, it has been demonstrated that the Sp5O projects onto higher centers involved in nociceptive information processing such as the thalamus [61], [62] and the parabrachial area [63]. Thus the trigeminal brainstem somatosensory system is an appropriate model to study the mechanisms of dynamic mechanical allodynia after disinhibition.

### Glycine inhibitory dysfunction gates Aβ input to superficial NS neurons

Although our initial hypothesis attempted to explain the development of tactile allodynia purely on the basis of changes in WDR neurons [64], we found that the hyperexcitability of WDR neurons disinhibited by application of strychnine in the Sp5O was not paralleled with cardiovascular responses comparable to those evoked by nociceptive stimuli [34]. This suggests that in such condition, low threshold input was not miscoded as nociceptive by WDR neurons. In contrast, our *in vivo* electrophysiological results show for the first time that strychnine disinhibition renders superficial lamina NS neurons sensitive to tactile stimuli applied to their receptive field, through indirect Aβ input gating. Consistent with these results, we found that strychnine application within the MDH superficial laminae also changed cardiovascular responses to innocuous stimuli, while increasing the excitability of downstream WDR neurons. Thus, alterations in neurons in the superficial lamina of the spinal cord play a key role in strychnine-induced dynamic mechanical allodynia.

Together with our electrophysiological findings, Fos data revealed that a normally silent circuit in the dorsal horn can simply and reversibly be recruited to activate neurons in the superficial laminae. Interestingly, Fos expression adopted a pattern involving not only the outer part of the superficial laminae, as previously found in other rat models of neuropathic pain [65], [66], but also the cellular region that overlaps the border between lamina II and III. Since in rats low threshold Aβ mechanoreceptive afferents can extend their arbors into inner lamina II [25], [41] as well as in laminas III-V, we hypothesized that a circuit may convey non-nociceptive information from here to NS superficial lamina neurons. Accordingly, Torsney and MacDermott [30] recently showed that in an immature rat spinal cord slice preparation there is a significant polysynaptic Aβ-fiber input to lamina I neurons and that lamina III neurons receive monosynaptic Aβ-fiber input. Since glycinergic neurons in laminas II and III receive a major monosynaptic input from myelinated primary afferents and stimulation of rat skin with brushing elicits a barrage of inhibitory postsynaptic currents at this site [67], we believe that under physiological condition, inhibitory drive mediated by glycinergic synapses onto excitatory interneurons in laminas II-III prevents the access of low-threshold input onto superficial lamina NS neurons. Accordingly, using in vivo intracellular recordings in anaesthetized rats, Weng and Dougherty [68] showed that brushing of the skin could provoke subthreshold excitatory potentials in dorsal horn NS neurons. After depolarization of the neurons, brushing in the original receptive field zone resulted in action potential firing. Thus, under pathological conditions or following removal of glycine inhibition, the circuit from lamina II-III to the superficial lamina could be activated. This would allow the transfer of Aβ input to structures integrating nociceptive information such as the parabrachial area, providing a mechanism by which touch turns into pain.

### Role of PKCγ

As shown by the results of the immunocytochemical study, the local circuit involved dorsal horn neurons expressing PKCγ. This enzyme has been well studied with regards to neuropathic pain. Increased expression of PKCγ is clearly documented in animal models of peripheral neuropathy [69], [70] as well as increased phosphorylation of cyclic AMP response element-binding protein in dorsal horn neurons expressing PKCγ [71]. Furthermore, mice that lack PKCγ almost completely failed to develop a neuropathic pain syndrome, including tactile allodynia, after partial sciatic nerve section [42]. The great majority of PKCγ cells are likely to be excitatory interneurons [72]. Interestingly, they have been found to receive direct Aβ-fiber input [73]. We show here that they are potentially under direct glycine receptor-mediated inhibition. They are thus adequate candidate to participate in the transfer of low-threshold input toward superficial lamina NS neurons in conditions of disinhibition. This occurs most probably polysynaptically, through additional interneurons [74], since the majority of PKCγ interneurons have axons and dendrites that arborize in the same layer as the cell body and do not reach the most superficial lamina [72].

In accordance with the suggested role of PKCγ interneurons, selective inhibition of segmental PKCγ with relevant doses of KIG31-1 [44], [75], [76] prevented the behavioral responses and nociceptive neuron excitation that develop after strychnine. KIG31-1 is not only a very selective *in vitro* inhibitor of PKCγ [45], [46]. The PKCγ inhibitor effects have also been characterized *in vivo*. It attenuates phase 2 responses of formalin-induced nociception in neonatal rats [44], prevents thermal hyperalgesia on morphine withdrawal [75] and attenuates ethanol withdrawal-associated mechanical allodynia [76]. Our results with the use of KIG31-1 further show that activation of PKCγ is a critical step in the profound alterations of nociceptive neuron responses to innocuous input following disinhibition. Interestingly, we found that KIG31-1 blocked Fos expression not only in lamina I neurons, but also in lamina II-III interneurons. That suggests that within PKCγ neurons, Fos expression happens downstream to PKCγ activation. Indeed, PKCγ translocation is an early phenomenon that can propagate very quickly, on a second time-scale, after appropriate stimulation [77]. Thus, as previously suggested [42], PKCγ in the dorsal horn could be a substantial target for preventing the development of tactile allodynia in the clinical setting.

### Role of NMDA receptors

We show here that blockade of NMDA receptors in the medullary dorsal horn can prevent development of strychnine-induced trigeminal tactile allodynia and activation of the underlying dorsal horn circuit as well. Such findings are consistent with previous demonstration that spinal administration of agents which antagonize the NMDA receptor reduces the behavioral, autonomic and electrophysiological hyperreactivity following intrathecal strychnine [19], [21], [78]. They are also in line with the regular demonstration that NMDA receptor activation is required for central sensitization in the dorsal horn [48]. PKCγ expressing interneurons could be involved in this mechanism. Indeed, PKCs, in particular PKCγ, potentiate NMDA receptor activity [79]–[81]. Such potentiation could result from phosphorylation of subunits of the NMDA receptor, as in other pain models [81], or from modulation of NMDA receptor trafficking and gating [80]. Since lamina I neurons seem to lack functional NMDA receptors [82] (but see references [83], [84] for divergent data) and d-AP5 blocked Fos expression not only in lamina I neurons, but also in lamina II-III interneurons, we would like to suggest that brush-activation of PKCγ under strychnine may open the gate to signaling in superficial dorsal horn neurons through activation of NMDA receptors within lamina II-III.

### Conclusion

To summarize, our current findings demonstrate the existence of a normally inactive dorsal horn circuit that produces dynamic mechanical allodynia when disinhibited. Although this could provide a source of neuropathic pain, we cannot dismiss the possibility that it is also at work in inflammatory pain. It would then act as a complementary mechanism to the protein kinase A dependent blockade of inhibitory glycine receptors by prostaglandin E2 that occurs in the superficial dorsal horns in response to peripheral inflammation [85], [86]. Finally, our results also suggest that alleviating dynamic mechanical allodynia could be attained through selective pharmacological inhibition of PKCγ, thus providing a new therapeutic tool for this disabling symptom.

## Material and Methods

### Animals

Adult male Sprague-Dawley rats (240–280 g) were obtained from Charles River (L'Arbresle, France) and maintained in a controlled environment (lights on 08:00-20:00, 22°C) with food and water freely available. All efforts were made to minimize the number of animals used. The experiments followed the ethical guidelines of the International Association for the Study of Pain and the European Community Council directive of 24 November 1986 (86/609/EEC).

### Chemicals

Unless specifically stated, all chemicals were obtained from Sigma. The selective PKCγ inhibitor KIG31-1 was obtained from Kai Pharmaceuticals Inc (San Francisco, CA). It is conjugated to Tat, a peptide carrier, via a cysteine-cysteine bond at its N terminus. KIG31-1 competes with activated PKCγ for binding to the isoenzyme-specific docking proteins, receptors for activated C kinase. This strategy prevents PKCγ translocation in an isoenzyme-specific manner [45], [47]. Linking of KIG31-1 to Tat enables efficient transfer of the peptide into cells [87].

### Intracisternal injections and behavioral testing

Immediately before the test stimulation session, rats were adapted to the observation field and red light for 30 minutes (habituation session). During this period, the experimenter reached into the cage to apply gentle air puffing on the face or the paw of the animal (see below). Animals were then briefly (<3 minutes) anesthetized using a mask with 2% halothane and received an intra-cisternal injection of either strychnine (10 µg in 5 µl aCSF) or aCSF alone (5 µl) using a 10 µl Hamilton syringe. aCSF consisted of: 150 mM Na^+^, 3 mM K^+^, 0.8 mM Mg^2+^, 1.4 mM Ca^2+^, 155 mM Cl^−^, pH 7.4, 295 mosmol.kg^−1^. Following recovery (<2 minutes), rats were placed in the observation field (0.6×0.6 m square) under red light for a 36-minute period test. Gentle air puffing (1 s-duration) was applied every 3 minutes onto the orofacial region or the hind paw using a calibrated pump by a first experimenter. Stimulation was carried out when the rat was in a sniffing/no locomotion state: with four paws placed on the ground, neither moving nor freezing. The distance to the target from which the stimulus was applied varied from 2 to 5 cm. The tip of the pump was moved towards the target from behind the animal so that it could not see it. Each series of stimulation consisted of 5 air puffs applied every 10 s.

The behavioral responses were observed and quantified by a second experimenter according to the method developed by Vos et al. [33]. A rat's response to mechanical stimulation consisted of one or more of the following elements: (1) detection, rats turn head toward stimulus; (2) withdrawal reaction, rats pull paw away or turn head away or pulls it briskly backward when stimulation is applied (a withdrawal reaction is assumed to include a detection element preceding the head or paw withdrawal and therefore consists of two responses elements) (see Table 2); (3) escape/attack, rats avoid further contact with the stimulus, either passively by moving their body away from the stimulus, or actively by attacking the tip of the pump; (4) asymmetric grooming, rats display an uninterrupted series of at least three wash strokes, licking or biting directed to the stimulated area.

**Table 2:** Response scoring system.

	Observed response elements				
Response category	Detection	Withdrawal	Escape/attack	Face grooming	Score
No response	0	0	0	0	0
Non aversive response	1	0	0	0	1
Mild aversive response	1	1	0	0	2
Strong aversive response	1	1	1	0	3
Prolonged aversive behavior	1	1	1	1	4

The following rank-ordered descriptive responses categories were formulated according to the study by Vos et al. [33]: no response, non aversive response, mild aversive response, strong aversive response, prolonged aversive behavior (Table 2). Each category was given a score (0–4) based on the number of observed response elements. According to Vos et al. [33], the score was assumed to reflect the magnitude of the aversiveness evoked by the mechanical stimulation. Score was equal to zero in case of absence of response. A mean score value was then calculated for each stimulation series.

For experiments investigating the effect of selective PKCγ inhibition, immediately before the habituation session rats received an intracisternal administration of KIG31-1 (1, 10, 100 or 500 pmoles, dissolved in 5 µl vehicle) or the vehicle alone (5 µl, Tat carrier), under brief halothane anesthesia.

For experiments investigating the motor effects of KIG31-1, rats were tested on the accelerating rotarod before and 30, 50 and 60 minutes after the inhibitor (500 pmoles) was injected intracisternally under brief halothane anesthesia.

For experiments investigating the effect of selective NMDA receptor inhibition, rats received an intracisternal administration of d-AP5 (10 µg, dissolved in 5 µl aCSF) or aCSF alone under brief halothane anesthesia 30 minutes before the habituation session.

### In vivo electrophysiology

As previously described [35], [36], animals were anesthetized with 2% halothane in a nitrous oxide/oxygen mixture (2:1). After intraperitoneal injection of 100 µg atropine sulfate, a tracheal cannula was inserted and the carotid artery and the jugular vein were cannulated. Animals were then paralyzed by an intravenous perfusion of pancuronium bromide (0.5 mg/h) and artificially ventilated with a volume-controlled pump (54–55 strokes/min). The levels of halothane, O_2_, N_2_O, and the end-tidal CO_2_ (3.5–4.5%) were monitored by an anesthetic gas monitor (Artema MM 200, Sundbyberg, Sweden) during the entire experimental period. The arterial catheter was attached to a calibrated pressure transducer (UFI, Morro Bay, CA) connected to an amplifier (Stoelting, Wood Dale, IL) for continuous monitoring of the mean arterial blood pressure (MAP). The analog output from the blood pressure amplifier was connected to a computer data sampling system (Cambridge Electronics Design 1401 computer interface; Cambridge, UK). Heart rate was monitored continuously and cutaneous vascularization was periodically checked by observing the color of the paw extremities and the rapidity by which they regained normal color after pressure application. Core temperature was maintained at 37±0.5°C with a homeothermic blanket system.

Animals were then placed in a stereotaxic frame with the head fixed in a ventroflexed position (incisor bar dropped 5 mm under the standard position) by means of an adapted metallic bar. For Sp5O recordings, a craniotomy was performed on the right side at the level of the occipito-parietalis suture and the dura mater was removed. For MDH recordings, the skin and muscles overlying the occipital bone were removed. A cervical laminectomy and suboccipital craniotomy were carried out to expose the medulla. The dura mater was then opened and the medulla was stabilized by a stainless-steel frame held in position with a micro-manipulator and a 2% Ringer-agar gel. After surgery, the level of halothane was reduced to 0.7–0.8% and maintained at this level during the recording period.

Unitary extracellular recordings were made using glass micropipettes (8–10 MΩ) filled with a mixture of 5% NaCl and pontamine sky blue in the Sp5O or the MDH superficial laminae (*i.e.* <150 µm below the medulla dorsal surface). Neurons were classified as WDR or NS based on their responses both to mechanical and percutaneous electrical stimulation applied to their receptive fields. Innocuous mechanical stimuli included air puffs, brushing with a soft brush, gentle stroking, and light pressure with a blunt probe. Nociceptive mechanical stimuli consisted of heavy pressure, pinprick, and pinching with fine forceps. Electrical square-wave stimuli (0.1–2 ms, 0.1–0.5 Hz) were applied through a pair of stainless steel stimulating electrodes inserted subcutaneously into the center of the previously delineated receptive field to determine whether the neuron was receiving input from A or C-fibers. Post-stimulus histograms were analyzed to distinguish responses due to A and C-fiber inputs, according to their latencies. The latency value of the responses was used to determine the conduction velocity of afferent inputs after making allowance for the conduction distance (∼50 mm) and 1 ms for the central synaptic delay, the delay in activation of the peripheral axons and the narrowing of afferents in the trigeminal spinal tract. Based on the distance to the recording site, responses with a latency of 2.5 ms (only seen for WDR neuron) or less were related to Aβ-fiber input, and latencies between 5 and 12 ms were related to Aδ input. Responses later than 30 ms after stimulation were classified as being related to C-fiber input. Thresholds were defined as the lowest value of intensity stimulation that elicited 1-2 spikes/trial at the corresponding latency in at least four to six trials.

Strychnine was administered within the Sp5O or the superficial laminae of the MDH through a glass micropipette connected to a Hamilton syringe (1 µl) by means of polyethylene tubing [35] during recordings from Sp5O neurons or intracisternally during recordings from MDH neurons. KIG31-1 was administered within the Sp5O through a glass micropipette connected to a Hamilton syringe (1 µl) by means of polyethylene tubing [35] during recordings from Sp5O neurons. Responses to mechanical stimulation of the skin were determined by applying brief (10 s) innocuous stimulation to the most sensitive portion of the cutaneous receptive field [36]. Innocuous stimuli consisted of slowly passing a soft bristled brush across the cutaneous receptive field (one 5–7 s brush stroke from caudal to rostral and one 5–7 s brush stroke from rostral to caudal). Only one neuron per rat was studied. Along with electrophysiological recordings, the magnitude of the MAP change (ΔMAP) was calculated by subtracting MAP before the onset of the stimulus from the peak response value (defined as the maximal increase in MAP during the period in which mechanical stimuli were applied).

At the end of the experiment, each recording site was marked by electrophoretic deposition of pontamine sky blue. After the animal was killed by injection of a lethal dose of pentobarbital, the brain was removed and fixed in a 10% formalin solution for one week. The tissue was frozen, cut into serial 100 µm thick sections and stained with neutral red. Recording sites (blue dye marks) were localized by microscopic examination and then plotted on camera lucida drawings of serial sections for further analysis.

### Immunocytochemistry and retrograde tracing

In a first group, rats were anaesthetized deeply with urethane (1.5 g/kg i.p.) as previously described [88]. Twenty minutes after the injection, the depth of the anesthesia was tested, and rats received an intra-cisternal injection of strychnine (40 µg in 5 µl aCSF) or vehicle (aCSF). This was immediately followed by innocuous stimulation of the right upper lip using a soft paintbrush for 10 minutes at 0.5 stroke/s. Two hours later, rats were perfuzed transcardially with warm (37°C) heparinized saline (25 IU heparin/ml) followed by cold (10°C) phosphate-buffered solution (0.1 M, pH 7.6) containing 4% paraformaldehyde and 0.03% picric acid for 15 minutes. The brainstem was then removed and transferred in the same paraformaldehyde-picric acid solution containing 30% sucrose at 4°C and left overnight. Coronal sections were cut on a freezing microtome and collected in a 0.05 M Tris-buffered saline (TBS). Free-floating brainstem sections were placed in 1% normal goat serum for 1 h before incubation at 4°C in a rabbit antibody directed against Fos (1∶1,000, 48 h, Oncogene Science, San Diego, CA), and a secondary goat antirabbit antibody (1∶400, 2 h). Immunoreactivity for Fos was visualized using nickel-DAB.

In a second group of animals, stimulated in the same conditions as the first group, brainstem sections underwent free-floating sequential immunostaining for Fos and PKCγ. Primary rabbit antibodies specific for PKCγ (1∶40,000, 48 h, Santa Cruz, CA) and a secondary goat antirabbit antibody (1∶400, 2 h) were used. Immunoreactivity for PKCγ was visualized using DAB only.

In a third group, rats were anaesthetized with chloral hydrate (10%, 400-mg/kg-body weight, i.p.) and placed in a stereotaxic frame. A glass micropipette containing the retrograde fluorescent tracer Fluorogold was inserted stereotaxically so that its tip (30–40 µm diameter) was located within the right parabrachial area (AP −8.8 mm to −9.8 mm, ML 2.0 to 2.4 mm, P 6.6 mm to 7.0 mm according to Paxinos and Watson [89]. Electrophoretic application of Fluorogold was made by 10 second pulses of positive direct current (3–5 µA) applied every 20 seconds for a period of 8–12 minutes. The microelectrode was left *in situ* for a further 5 minutes before withdrawal from the brain. One single application was made in the parabrachial area per animal. One week later, rats were anaesthetized with urethane and then stimulated in the same conditions as the first group. Brainstem sections underwent free-floating sequential immunostaining for Fos and Fluorogold. Primary rabbit antibodies specific for Fluorogold (1∶6,000, 48 h, Chemicon, CA) and a secondary goat antirabbit antibody (1∶200, 2 h) were used. Immunoreactivity for Fluorogold was visualised using DAB only.

In a fourth group, animals were not stimulated and brainstem sections underwent free-floating immunofluorescent staining for the glycine receptor α and β subunits and PKCγ using a mouse monoclonal primary antibody (1∶200, Synaptic Systems, Göttingen, Germany) and a rabbit primary antibody (1∶5,000, Santa Cruz, CA) respectively. The incubation lasted 24 h at room temperature. Immunoreactivity was revealed using Cy3 conjugated goat anti-mouse (1∶400) and Cy2 conjugated goat anti-rabbit (1∶200) secondary antibodies respectively (1 h at room temperature).

In a fifth group, rats were treated as in group 1 but received an intracisternal injection of KIG31-1 (500 pmoles in 5 µl vehicle) or vehicle alone (Tat carrier peptide) 20 minutes after induction of anesthesia. Strychnine was given 30 minutes later.

In a sixth group, rats were treated as in group 1 but received an intracisternal injection of d-AP5 (10 µg in 5 µl aCSF) or aCSF alone 20 minutes after induction of anesthesia. Strychnine was given 1 hour later.

In all cases, sections were rinsed in TBS several times between and after each incubation and finally transferred onto gelatinized slides before being coverslipped using DPX. All immunolabels were diluted in TBS containing 0.25% bovine serum albumin and 0.3% Triton X-100. Specificity controls consisted of the omission of the primary antibody and incubation of sections in inappropriate secondary antibodies. In all these control experiments, no specific staining was evident. A few selected sections were mounted separately and slightly counterstained with cresyl violet to help delineate the limits of the anatomical structures.

For data analysis, computer-assisted bright-field images of injection sites and representative labeling were obtained using a CCD color video camera (Sony DXC-950P) connected to a Nikon Optiphot-2 microscope. Images were exported to Adobe PhotoShop (v 5.5) to adjust brightness and contrast before adjusting the image scale. Each injection site was analyzed using coronal sections processed with DAB. Representations of the sites of injection were grouped on standard drawings of the parabrachial area. PKCγ and glycine receptor immunofluorescence was analyzed using a motorized Zeiss Axioplan 2 Imaging microscope coupled with a Hamamatsu C4742-95 digital camera, by switching between fluorescein and Texas Red filter sets. For illustrations, numerical images of immunofluorescence were deconvoluted using Metamorph 5.4 software. In each rat, Fos immunoreactive nuclei, immunopositive PKCγ or glycine receptor neurons, retrogradely and dual labeled neurons were counted according to their location in the different laminae of the MDH from 5 different sections, each taken at a given rostrocaudal plane within the MDH. Intervals of 400 µm between planes ensured that cells were counted only once. The delineation of the MDH was based upon the Paxinos and Watson atlas [89] and our own myeloarchitectural atlas. The data is expressed as the sum of the total number of labeled cells counted from all 5 sections that were analyzed in each animal.

### Statistical analysis

For data analysis one-way ANOVA for repeated measures followed by Dunnett's or Newman-Keuls tests were used and the level of significance was set at *P*<0.05. Results are expressed as mean±SEM.
